# Supplementary material for: Subsidence more than doubles sea-level rise today along densely populated coasts
Source: Nat Commun. 2026 May 16;17:4382. doi: 10.1038/s41467-026-72293-z (PMC13179951; doi:10.1038/s41467-026-72293-z)
Supplement: Supplementary file 1 — Supplementary Information [file 41467_2026_72293_MOESM1_ESM.pdf]

Supplementary Material to:

Subsidence more than doubles sea-level rise today along densely populated coasts

Author Information

Julius Oelsmann[1,2], Robert J. Nicholls[3,4], Daniel Lincke[5], Marta Marcos[6], Manoochehr Shirzaei[7], Laura Sánchez[1], Leonard Ohenhen[8], Denise Dettmering[1], Jochen Hinkel[5,9], Benjamin P. Horton[10], Florian Seitz[1]

[1] Deutsches Geodätisches Forschungsinstitut, Technische Universität München, Arcisstraße 21 80333 München, Germany;
[2] Department of River-Coastal Science and Engineering, Tulane University, 6823 St. Charles Avenue New Orleans, LA 70118, USA, Email: joelsmann@tulane.edu
[3] Tyndall Centre for Climate Change Research, University of East Anglia, Norwich, UK,
[4] School of Engineering, University of Southampton, Southampton, SO17 1BJ, UK
[5] Global Climate Forum, Neue Promenade 6, 10178 Berlin, Germany
[6] IMEDEA, (UIB-CSIC), Miquel Marquès, 21, Esporles, 07190, Balearic Islands, Spain
[7] Department of Geosciences, Virginia Tech, Blacksburg, VA, USA.; Virginia Tech National Security Institute, Virginia Tech, Blacksburg, VA, USA. Institute for Water, Environment and Health, United Nations University, Hamilton, Ontario, Canada.
[8] University of California, Department of Earth System Science, Irvine, CA, USA
[9] Resource Economics Group, Albrecht Daniel Thaer-Institute and Berlin Workshop in Institutional Analysis of Social-Ecological Systems (WINS), Humboldt-Universität zu Berlin, Germany
[10] School of Energy and Environment, City University of Hong Kong, Hong Kong SAR

Correspondence to: *Julius Oelsmann, joelsmann@tulane.edu

This file includes:

Figures S1 to S9

Table S1 to S2

SI data files

SI References

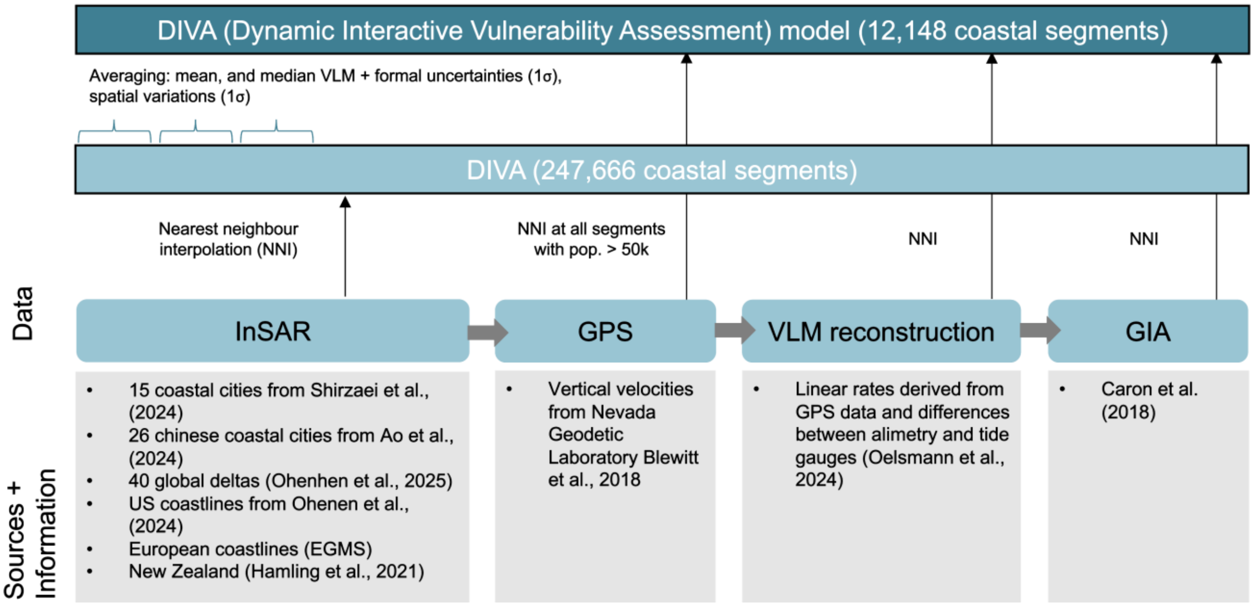

Figure S1: Workflow of integrating different datasets into the DIVA (Dynamic Interactive Vulnerability Assessment) model frame work. All datasets are mapped onto the DIVA model segments using nearest neighbour interpolation. The coastal segments are 'filled' from left to right, e.g., we incorporate all InSAR data wherever available, then GNSS data in cities is used where no InSAR data is available, then the remaining segments are filled with the VLM reconstruction (OE24) and the GIA model (Caron et al., 2018).

**Dataset**	**Europe EGMS**	**USA (Ohenhen et al., 2024)**	**Delta (Ohenhen et al., 2025)**	**Cities (Shirzaei et al., 2024)**	**Chinese cities (Ao et al., 2024)**	**New Zealand (Hamling et al., 2022)**
**Period**	2015-2021	2007-2020	2014-2023	2015-2020	2015-2022	2003-2011
**Mission**	Sentinel-1	Sentinel-1	Sentinel-1	Sentinel-1	Sentinel-1	Envisat
**Resolution**	100 m grid	50 m	1000 m	50 m	40 m	~ 1000 m
**Validation information**	weighted standard deviation: 1.19 mm/year (n=1617, NGL)	weighted standard deviation: 1.43 mm/year (n=943, NGL)	weighted standard deviation: 1.2 mm/year (n=81, NGL), as reported by Ohenhen et al., 2025	weighted standard deviation: 1.72 mm/year (n=119, NGL)	1.5 mm/year (RMSE) in comparison with CMONOC GNSS trends (n=32), as reported by Ao et al., 2024	1.6 mm/year standard deviation w.r.t. GNSS trends as reported by Hamling et al., 2021

Table S1: Information on the different InSAR datasets used in this research.

Validation

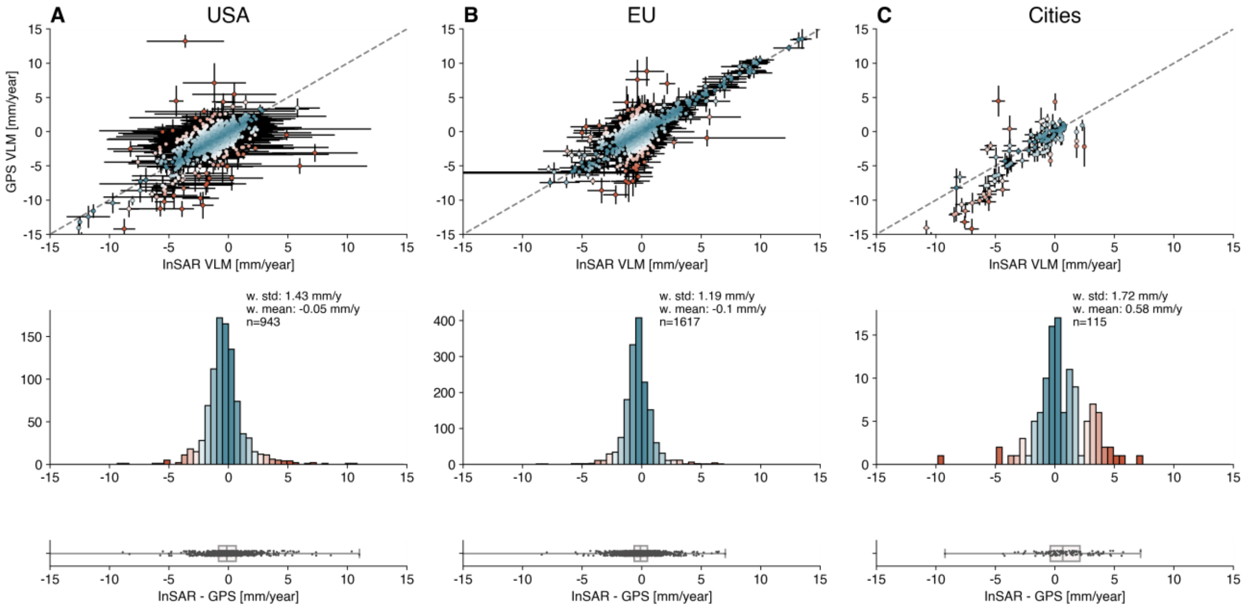

Figure S2: Comparison of vertical velocities of NGL GPS rates and the US (Ohenen et al., 2024), European (EGMS), and coastal city datasets (Shirzaei et al., 2024). Shown are scatter plots (where error bars indicate the individual formal uncertainties), and histograms, in which absolute differences are color-coded from (-5 - 5 mm/year). In the second row we show the weighted standard deviations and means, using the inverse of the square-root-sum of the squared uncertainties as weights. The EGMS InSAR data are transformed using a latitude($\boldsymbol{\phi}$)-dependent transfer function $\Delta V_{fit} = -2 \times 10^{-4}\phi^2 + 0.04\phi - 0.85$, defined by Thieblemont et al., 2024. To match the InSAR points with the GPS data, we used the weighted average of the data within a radius of 5 km, with inverted formal uncertainties as weights. The last row shows boxplots of the differences (InSAR minus GPS), including the median (central line), interquartile range (box; 25th–75th percentiles), and whiskers that extend to 1.5× of the interquartile range.

Local vertical land motion

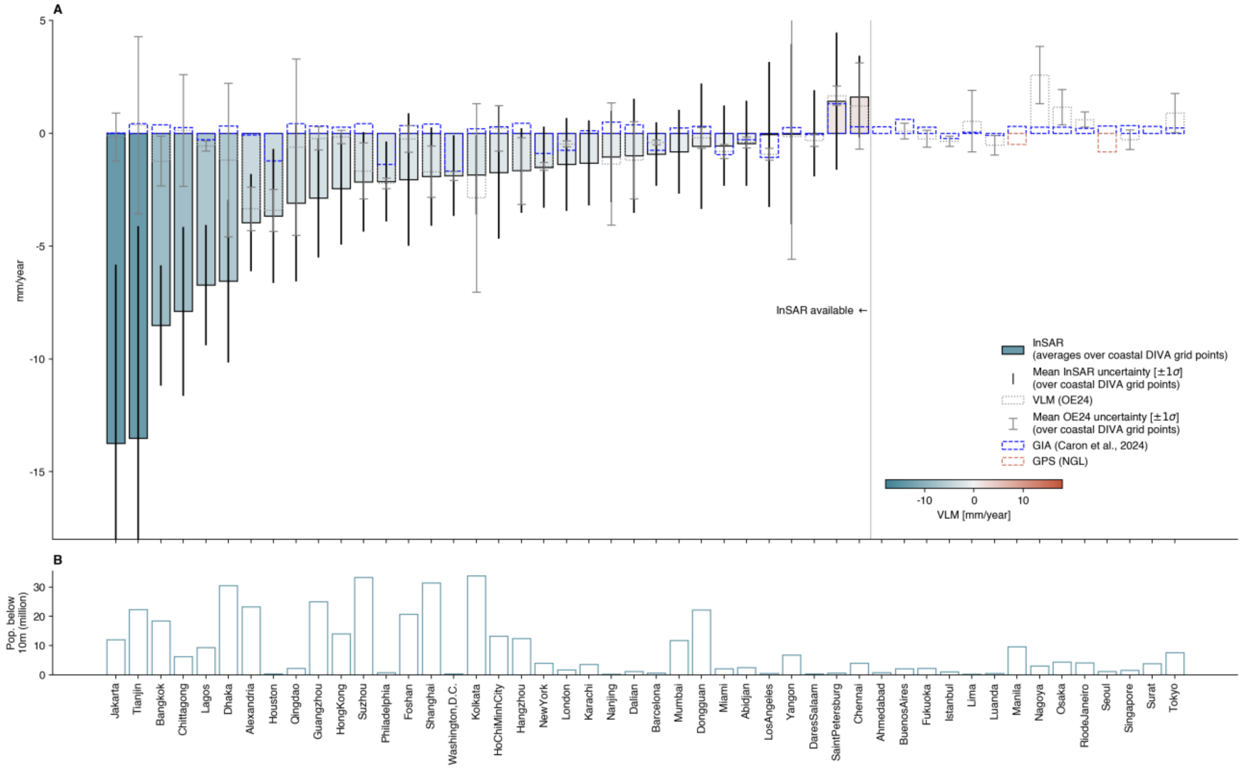

Figure S3: A Vertical land motion at different coastal cities. Shown are averaged rates provided at the coastal segments of the DIVA model within a radius of 50 km around the city area (which is here defined as the area that is covered by the InSAR dataset from Tay et al., 2022). Shown are the mean VLM values for InSAR (filled bars), the VLM reconstruction (bars with dotted outlines, OE24), GIA (bars with blue dashed outlines) and GPS from NGL, or Blewitt et al., 2016 (bars with red dashed outlines), where available. The thin black lines and grey lines indicate the mean 1σ uncertainties (Methods) for the InSAR data (i.e., the combined spatial, formal, and cross-validation) and the VLM reconstruction (OE24) for each city. **B** shows the averaged population at the same coastal segments.

National relative sea level change and vertical land motion

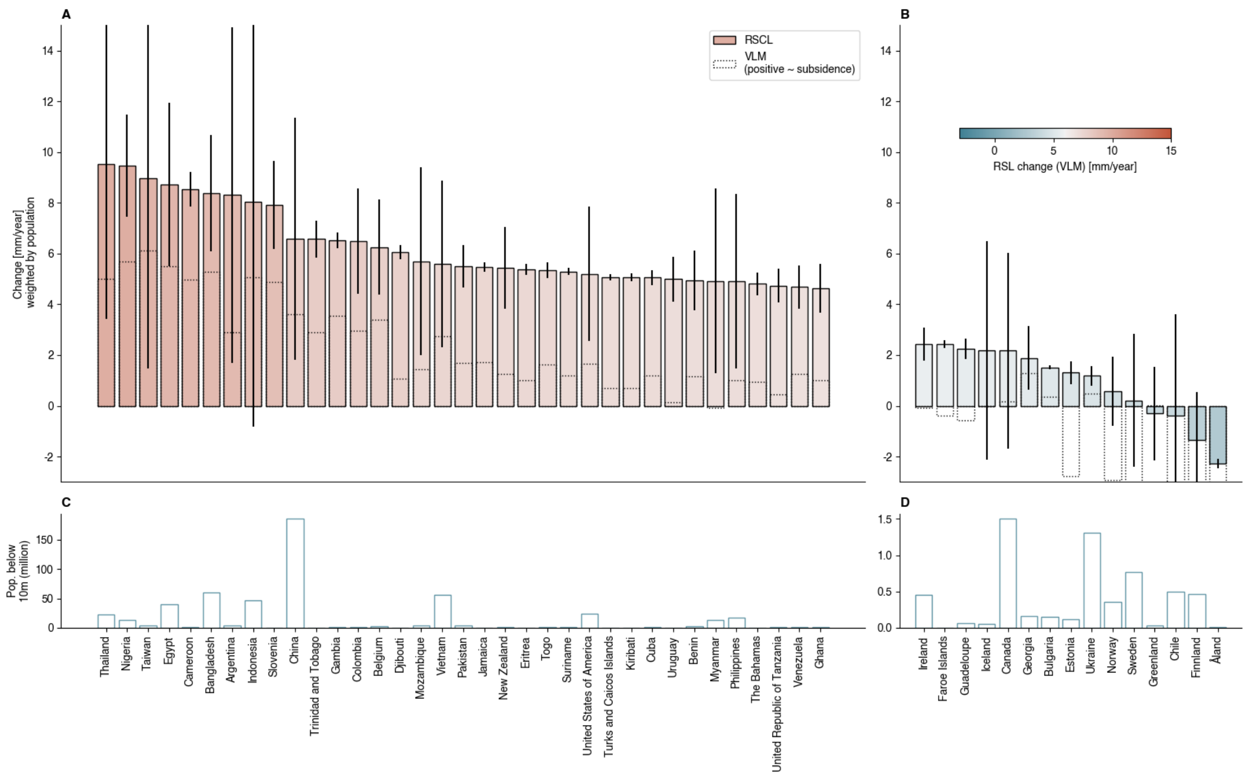

Figure S4: A, B Population weighted RSLC (filled bars) and weighted standard deviations (black lines) for different countries. The data is sorted such that **A** and **B** show a fraction of countries with the highest (lowest) population-weighted RSLC. The unfilled bars denote the population weighted VLM. **C, D** shows the averaged coastal population.

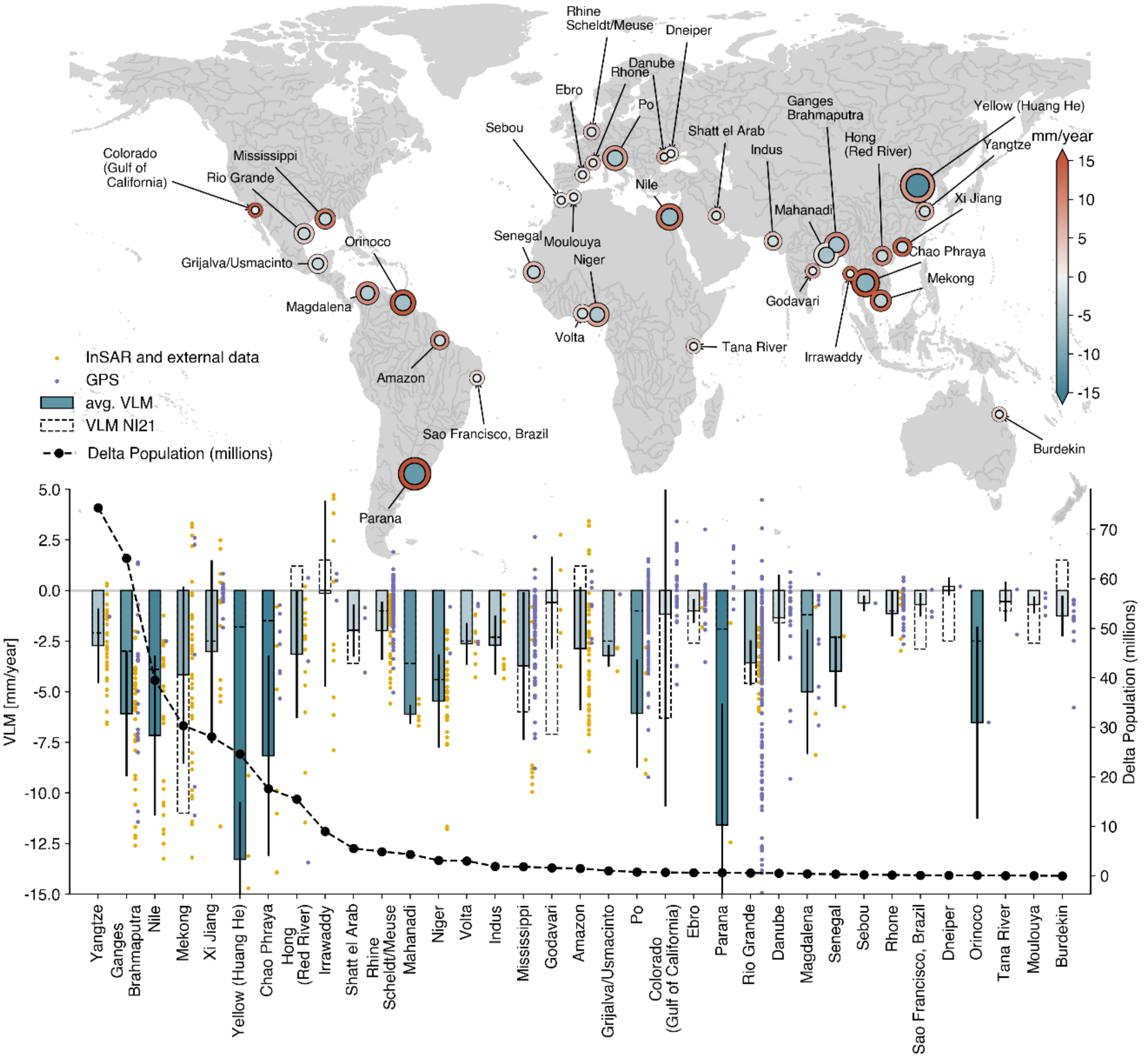

Figure S5: Statistics for 35 of the largest global deltas (according to a selection of the deltas discussed by Ericson et al., 2005). Shown are the geographical locations of the deltas, where the inner circles indicate the VLM averages in mm/year, which are either based on the averages of the combination of InSAR and GNSS data points (interpolated on the DIVA segments), or on one of the individual datasets, if the other is not available, see also SI data file 1). The outer circles represent the standard deviations of the VLM estimates per delta. These delta VLM-averages (and standard-deviations) are also illustrated by the filled bars (incl. errorbars) in the lower plot. The dashed bars indicate the delta-averages of NI21b. The yellow and blue dots indicate the individual InSAR and GNSS data points in the vicinity of the delta (i.e., the InSAR and GNSS data interpolated onto the DIVA grid).

Table S2: Population weighted RSLC and VLM (positive values denote subsidence) per country.

Country	RSLC [mm/year]	VLM [mm/year] (positive ~ subsidence)
Thailand	9.52	5.02
Nigeria	9.46	5.70
Taiwan	8.97	6.12
Egypt	8.72	5.50
Cameroon	8.53	4.97
Bangladesh	8.38	5.28
Argentina	8.31	2.89
Indonesia	8.03	5.05
Slovenia	7.91	4.89
China	6.59	3.62
Trinidad and Tobago	6.57	2.91
Gambia	6.53	3.54
Colombia	6.49	2.97
Belgium	6.25	3.38
Djibouti	6.06	1.07
Mozambique	5.69	1.45
Vietnam	5.59	2.76
Pakistan	5.50	1.69
Jamaica	5.47	1.71
New Zealand	5.45	1.25
Eritrea	5.37	1.01
Togo	5.35	1.64
Suriname	5.29	1.21
United States of America	5.19	1.66
Turks and Caicos Islands	5.08	0.70
Kiribati	5.07	0.69
Cuba	5.05	1.19
Uruguay	4.99	0.13
Benin	4.94	1.17
Myanmar	4.93	-0.08
Philippines	4.91	1.02
The Bahamas	4.81	0.94
United Republic of Tanzania	4.74	0.44
Venezuela	4.68	1.25
Ghana	4.64	1.01
Italy	4.62	1.88
Netherlands	4.55	1.71
Mexico	4.54	1.06
British Virgin Islands	4.52	0.95
Marshall Islands	4.52	0.60
...	...	...
Ireland	2.44	-0.09
Faroe Islands	2.43	-0.39
Guadeloupe	2.25	-0.57
Iceland	2.19	-0.01

Canada	2.17	0.16
Georgia	1.89	1.29
Bulgaria	1.51	0.35
Estonia	1.31	-2.78
Ukraine	1.18	0.49
Norway	0.57	-2.94
Sweden	0.22	-3.20
Greenland	-0.31	0.01
Chile	-0.40	-3.19
Finnland	-1.34	-5.29
Åland	-2.28	-6.32

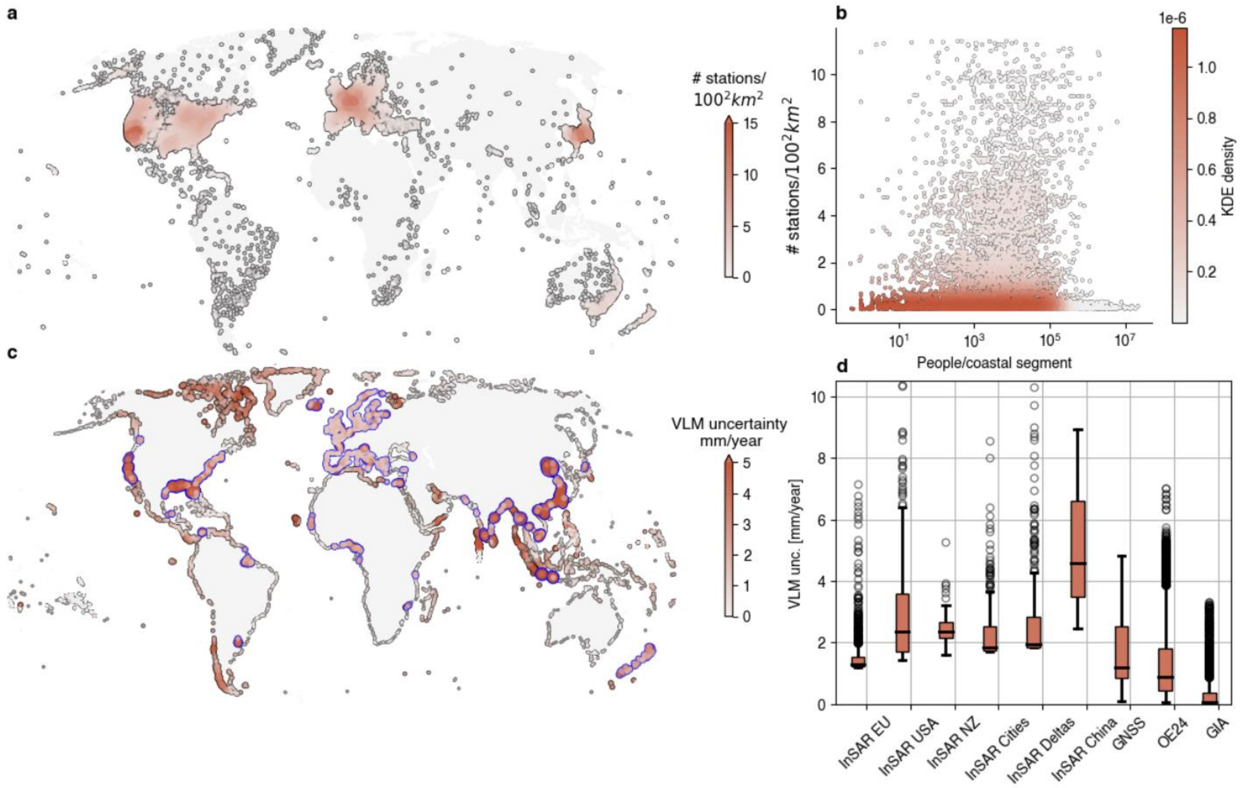

Figure S6: a**)** shows the station density in terms of number of stations per 100^2 km. The station density was computed based on the number of GNSS stations (from NGL) within a 500 km search radius around an individual station, divided by the area covered by this circle. **b)** compares the station density versus population (on a logarithmic scale). Colorbar shows the kernel density estimate (KDE), indicating how densely points cluster in the (population, station density) space. The values of the station density and the population (defined at the coastlines) are coupled by nearest neighbor interpolation. **c)** shows the VLM uncertainties, the InSAR and GPS data are highlighted by blue outlines. The boxplots in **d)** present the VLM uncertainties of the different dataset components. Note that these uncertainties are based on the combined formal, spatial and cross-validation uncertainties in the InSAR datasets (and the provided uncertainties in OE24, GNSS (Blewitt et al., 2018), and Caron et al., 2018 (GIA)).

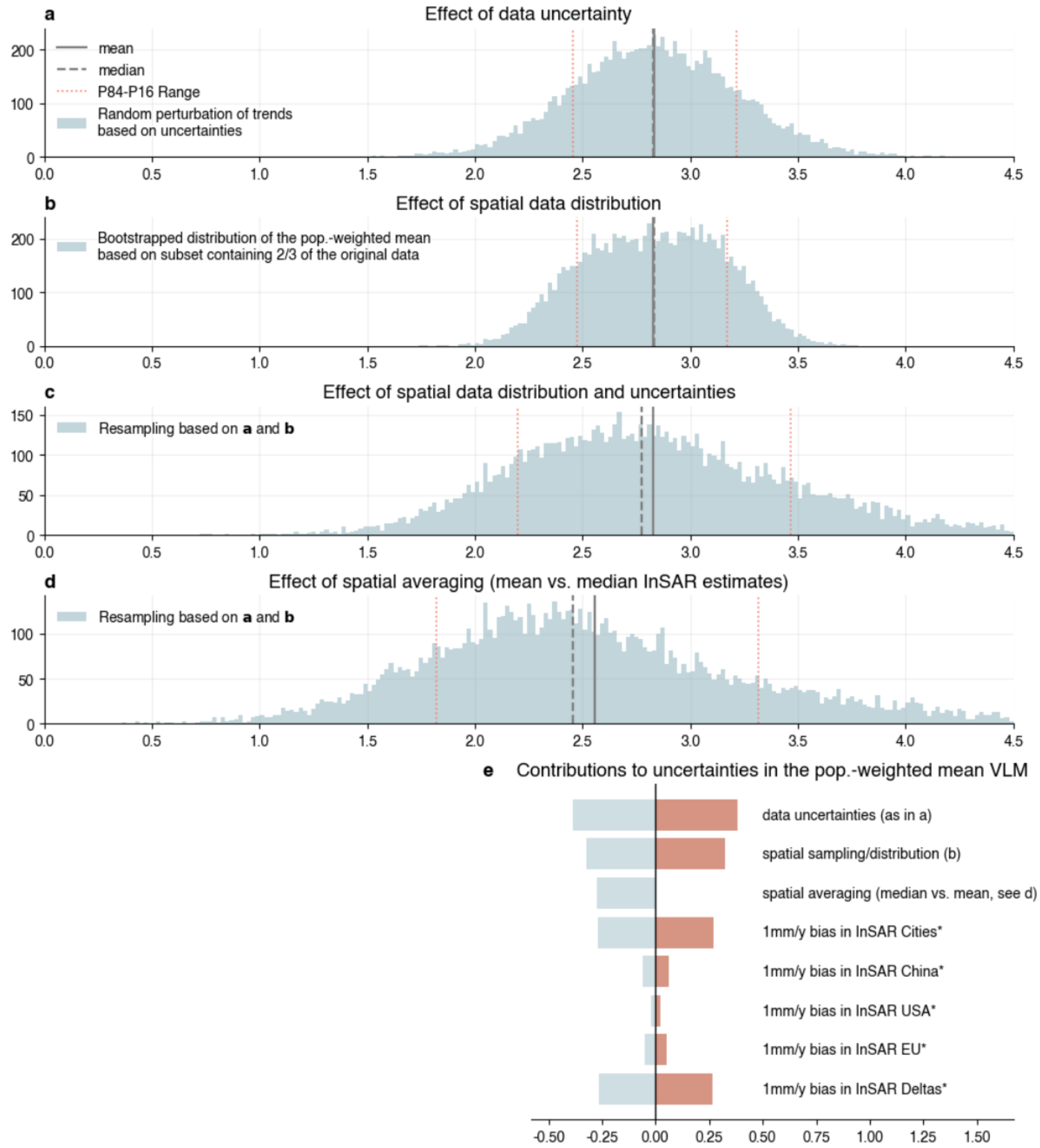

Figure S7: Contributions to uncertainties in the global population-weighted global VLM estimates. a) VLM values are perturbed using normally distributed random errors (i.e., combined formal, cross-validation, and spatial uncertainties, see Methods) over 10,000 iterations. Shown are the resulting distributions together with the mean (solid lines), median (dashed lines), and the 15.9–84.1 percentile range (red dotted lines, corresponding to ±1 standard deviation for a normal distribution). b) Uncertainty arising from spatial heterogeneity is estimated by repeatedly computing population-weighted mean VLM from 10,000 random subsamples containing 50% of the original data. c shows the combined effects of subsampling and random perturbations. d) Distributions obtained when using *median* InSAR rates (instead of means, as shown in a-c) during the aggregation from high-resolution InSAR to the coarser DIVA grid, based on the same subsampling and perturbation approach as in panels a and b. e) Relative contributions of the different uncertainty sources to the uncertainty in the mean. We additionally illustrate how *hypothetical systematic biases of ±1 mm yr^{-1} in the dataset would affect the global mean VLM estimate, highlighting the sensitivity of the results to potential measurement biases.

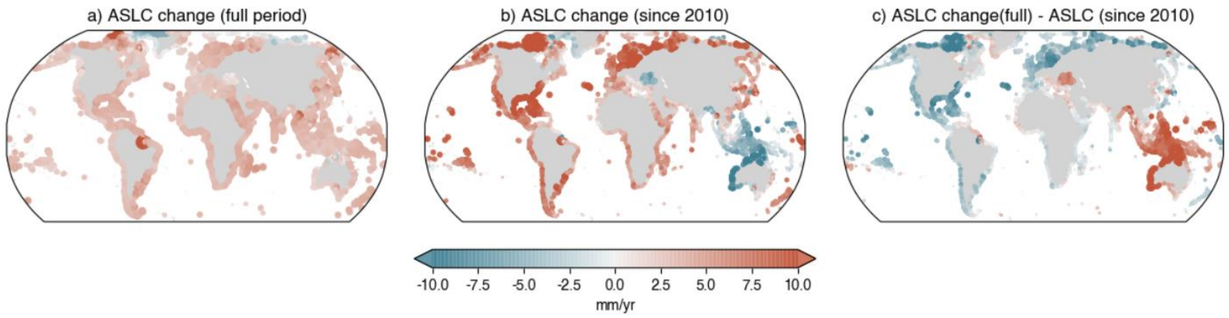

Figure S8: Absolute sea level changes derived over different periods. Shown are (a) coastal trends [mm/year] derived over 1995-2020, over 2010-2020 (b), as well as the differences between the two periods (c).

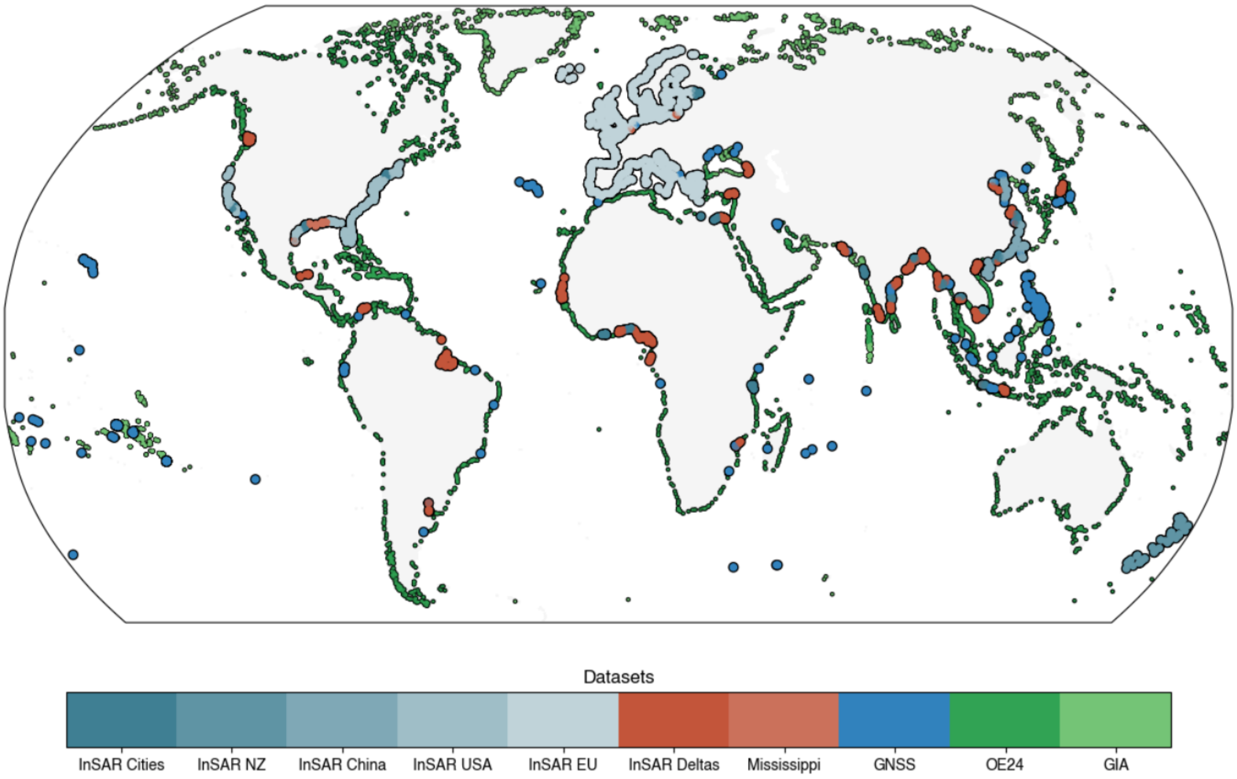

Figure S9: Global distribution of the underlying datasets used in the VLM estimate.

SI data file

The delta and city subsidence and the total global estimates can be downloaded from the zenodo repositories listed in 'data availability' in the main file.

SI References

1. Ao, Zurui, Hu, Xiaomei, Tao, Shengli, Hu, Xie, Wang, Guoquan, Li, Mingjia, Wang, Fang, Hu, Litang, Liang, Xiuyu , Xiao, Jingfeng, Yusup, Asadilla, Qi, Wenhua, Ran, Qinwei, Fang, Jiayi, Chang, Jinfeng, Zeng, Zhenzhong, Fu, Yongshuo, Xue, B.-L, Wang, Ping, Fang, Jingyun. (2024). A national-scale assessment of land subsidence in China's major cities. Science (New York, N.Y.). 384. 301-306. 10.1126/science.adl4366.
2. Blewitt, G., Kreemer, C., Hammond, W. C., and Gazeaux, J. (2016). Midas robust trend estimator for accurate gps station velocities without step detection. *Journal of Geophysical Research: Solid Earth*, 121(3):2054–2068.
3. Caron, L., Ivins, E. R., Larour, E., Adhikari, S., Nilsson, J., and Blewitt, G. (2018). Gia model statistics for grace hydrology, cryosphere, and ocean science. *Geophysical Research Letters*, 45(5):2203–2212.
4. European Ground Motion Service (EGMS), 2022, https://doi.org/10.2909/0a94b5d4-b414-4f2b-a6be-eea73094a0f5
5. Oelsmann, J., Marcos, M., Passaro, M., Sanchez, L., Dettmering, D., and Seitz, F. (2024). Vertical land motion reconstruction unveils nonlinear effects on relative sea level. *Nature Geosciences*
6. Ohenhen, L.O., Shirzaei, M., Ojha, C. et al. Disappearing cities on US coasts. Nature 627, 108–115 (2024). https://doi.org/10.1038/s41586-024-07038-3
7. Tay, C., Lindsey, E. O., Chin, S. T., McCaughey, J. W., Bekaert, D., Nguyen, M., Hua, H., Manipon, G., Karim, M., Horton, B. P., Li, T., and Hill, E. M. (2022). Sea-level rise from land subsidence in major coastal cities. *Nature Sustainability*.
8. Thiéblemont, R., Le Cozannet, G., Nicholls, R. J., Rohmer, J., Wöppelmann, G., Raucoules, D., de Michele, M., Toimil, A., and Lincke, D., Current State of Coastal Subsidence in Europe Derived from the European Ground Motion Service, submitted to Earth’s Future, 2024
